# Apoptotic Effect of Isoimpertorin via Inhibition of c-Myc and SIRT1 Signaling Axis

**DOI:** 10.3390/ijms25084248

**Published:** 2024-04-11

**Authors:** Hwan-Joo Ko, Su-Yeon Park, Deok Yong Sim, Sung-Hoon Kim, Soyoung Hur, Jang-Hoon Lee, Youngchul Kim

**Affiliations:** Department of Clinical Korean Medicine, Graduate School, Kyung Hee University, Seoul 02447, Republic of Korea; wellsleeping@naver.com (H.-J.K.); waterlilypark@naver.com (S.-Y.P.); simdy0821@khu.ac.kr (D.Y.S.); sngkim7@khu.ac.kr (S.-H.K.); shur3423@gmail.com (S.H.); komclive@khmc.or.kr (J.-H.L.)

**Keywords:** isoimperatorin, apoptosis, subG1 phase arrest, c-Myc, SIRT1

## Abstract

Though Isoimperatorin from *Angelicae dahuricae* is known to have antiviral, antidiabetic, anti-inflammatory and antitumor effects, its underlying antitumor mechanism remains elusive so far. Hence, the apoptotic mechanism of Isoimperatorin was explored in hepatocellular carcinomas (HCCs). In this study, Isoimperatorin inhibited the viability of Huh7 and Hep3B HCCs and increased the subG1 apoptotic portion and also abrogated the expression of pro-poly-ADP ribose polymerase (pro-PARP) and pro-caspase 3 in Huh7 and Hep3B cells. Also, Isoimperatorin abrogated the expression of cyclin D1, cyclin E1, CDK2, CDK4, CDK6 and increased p21 as G1 phase arrest-related proteins in Huh7 and Hep3B cells. Interestingly, Isoimperatorin reduced the expression and binding of c-Myc and Sirtuin 1 (SIRT1) by Immunoprecipitation (IP), with a binding score of 0.884 in Huh7 cells. Furthermore, Isoimperatorin suppressed the overexpression of c-Myc by the proteasome inhibitor MG132 and also disturbed cycloheximide-treated c-Myc stability in Huh7 cells. Overall, these findings support the novel evidence that the pivotal role of c-Myc and SIRT1 is critically involved in Isoimperatorin-induced apoptosis in HCCs as potent molecular targets in liver cancer therapy.

## 1. Introduction

Liver cancer ranks as the fifth fatal malignancy in humans, generally due to cirrhosis or inflammation [1,2]. Among liver cancers, hepatocellular carcinoma (HCC) is the most common type (over 90%) of liver cancer compared to other types of liver cancer, including intrahepatic cholangiocarcinoma and hepatoblastoma [3]. Also, secondary metastatic liver cancer from colon, lung and breast cancers is more common than primary liver cancer from the liver cells [4]. However, though sorafenib as a kinase inhibitor has been applied for late-stage liver cancer, its antitumor efficacy still remains unsuccessful to date. Hence, recently, molecular targeted therapies with natural compounds are attractive for liver cancer therapy [5].

c-Myc, one of driver oncogenes as one of the Myc family, is most tumorigenic with β-catenin, SmoM2, and short hairpin RNA targeting P53 (shp53), only when RAS signaling is activated in HCCs [6]. Hence, c-Myc is regarded a pivotal target for cancer treatment [7,8].

Sirtuin 1 (SIRT 1) plays a critical role in the progression of alcoholic and nonalcoholic fatty liver diseases by modulating hepatic lipid metabolism, hepatic oxidative stress and inflammation among seven mammalian sirtuins [9]. Recently, SIRT 1 has also been involved in cancer progression in concert with NFKB [9], HIF1α [10] and STAT3 [11]. Also, it is noteworthy that SIRT1 has been considered as a target of several diseases such as aging [12], cancer [13], autoimmune disease [14] and obstructive pulmonary disease [15].

Isoimperatorin isolated from *Angelicae dahuricae* [16] is known to have antiviral [17], antidiabetic [18,19], anti-inflammatory [19,20] and antitumor effects in colon [21], liver [22] and stomach [22,23] cancers. Nonetheless, the underlying molecular mechanisms are not fully understood yet. Thus, in the present study, the antitumor mechanism of Isoimperatorin was elucidated in Huh7 and Hep3B hepatocellular carcinomas in relation to the c-Myc/SIRT1 signaling axis.

## 2. Results

### 2.1. Isoimperatorin Inhibits the Viability of Huh7 and Hep3B Cells

**Figure 1:**
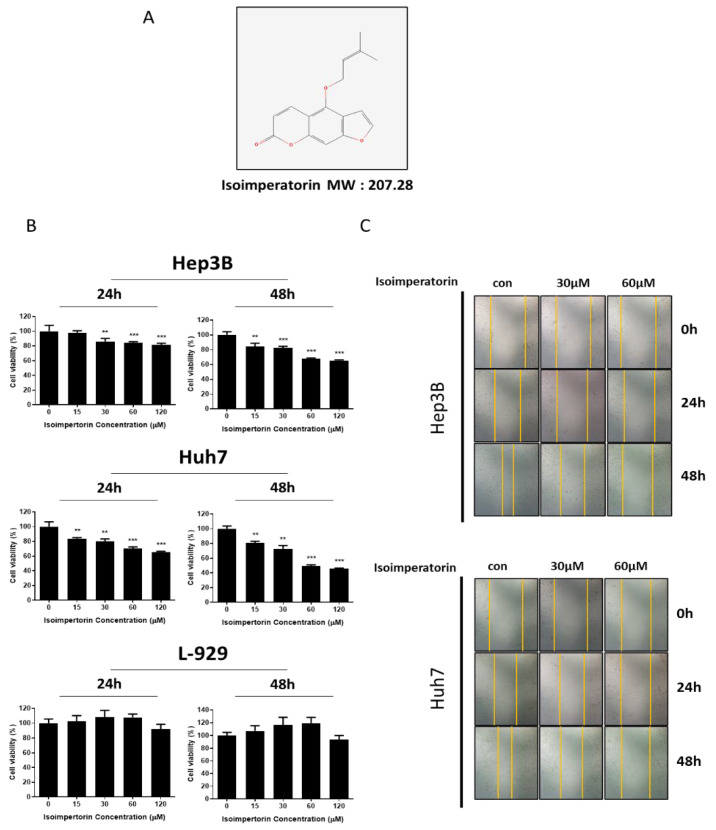
Effect of Isoimperatorin on the viability and migration of Huh7 and Hep3B cells. (**A**) Structure and molecular weight of Isoimperatorin; (**B**) effect of Isoimperatorin on the viability of Huh7, Hep3B and L-929 cells by MTT assay. ** *p* < 0.01, *** *p* < 0.001 vs. untreated control. (**C**) Effect of Isoimperatorin on the migratory activity of Huh7 and Hep3B cells by wound-healing assay.

Isoimperatorin (Figure 1A) significantly suppressed the viability of Huh7 and Hep3B cells in a concentration- and time-dependent manner without hurting L-929 cells compared to the untreated control (Figure 1B). Also, Isoimperatorin diminished the migratory effect in Huh7 and Hep3B cells by wound-healing assay.

### 2.2. Isoimperatorin Increases Sub G1 Population in Huh7 and Hep3B Cells

**Figure 2:**
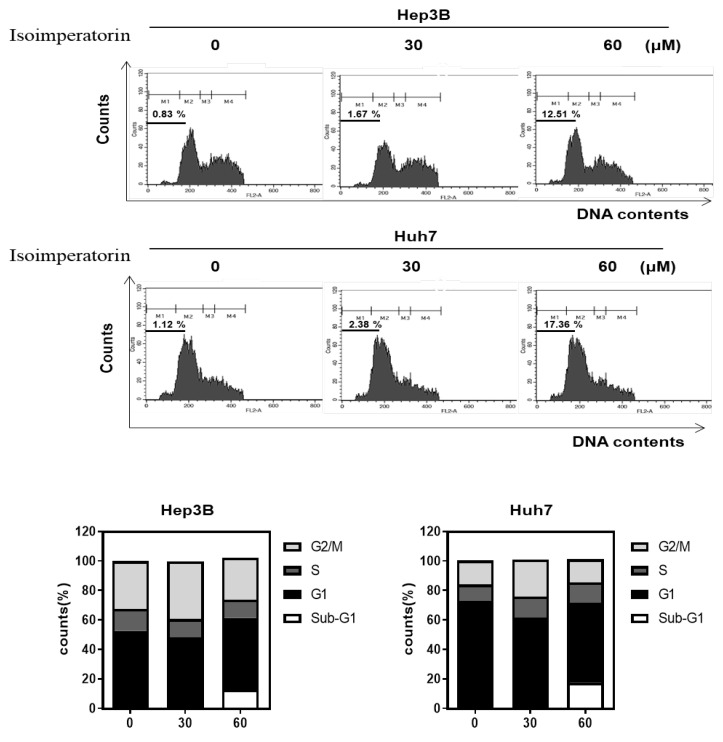
Effect of Isoimperatorin on sub G1 population in Huh7 and Hep3B cells. Huh7 and Hep3B cells were exposed to Isoimperatorin (30 and 60 μM) for 24 h and were subjected to cell cycle analysis.

Isoimperatorin significantly enhanced the apoptotic sub G1 population to 12.51% and 17.36%, respectively, in Huh7 and Hep3B cells compared to the untreated control (0.83%, 1.12%), in a concentration-dependent manner (Figure 2).

### 2.3. Isoimperatorin Attenuates the Expression of Pro-PARP and Pro-Caspase 3 in Huh7 and Hep3B Cells

**Figure 3:**
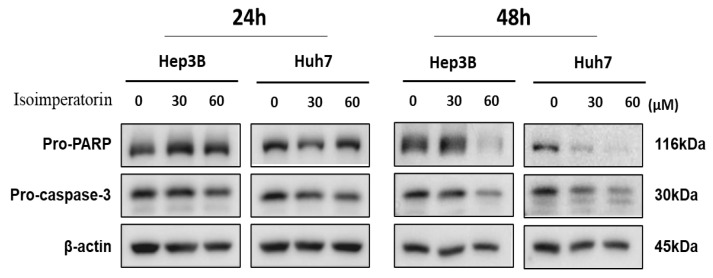
Effect of Isoimperatorin on pro-PARP and pro-caspase 3 in Huh7 and Hep3B cells. Huh7 and Hep3B cells were treated with Isoimperatorin (30, 60 μM) for 24 h and subjected to Western blotting.

Isoimperatorin reduced the expression of pro-PARP and pro-caspase 3 in a concentration- and time-dependent fashion in Huh7 and Hep3B cells (Figure 3).

### 2.4. Isoimperatorin Modulates Cell Cycle- and Survival-Related Proteins in Huh7 and Hep3B Cells

**Figure 4:**
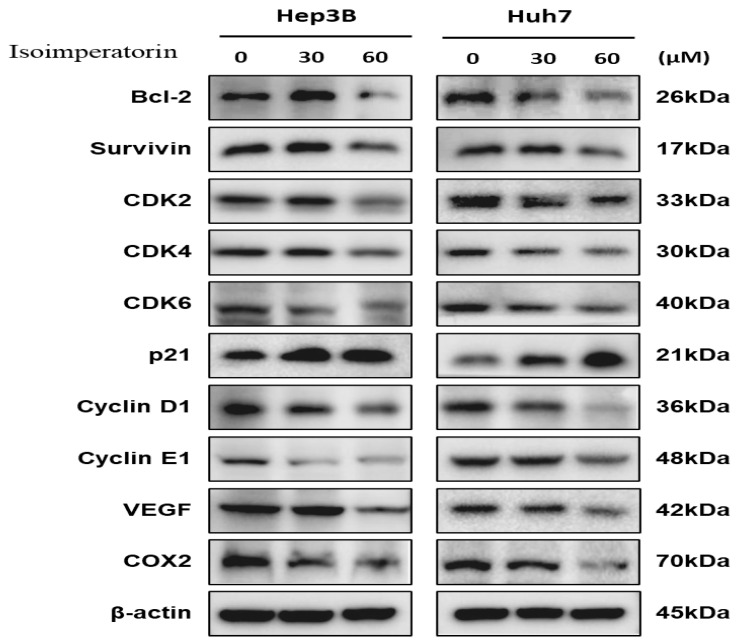
Effect of Isoimperatorin on cell cycle and survival-related proteins in Huh7 and Hep3B cells. Huh7 and Hep3B cells were treated with Isoimperatorin (30 and 60 μM) for 24 h and subjected to Western blotting.

Isoimperatorin abrogated the expression of CDK2, CDK4, CDK6, cyclin D1, cyclin E1 and activated p21 as cell cycle-related proteins in a concentration-dependent fashion in Huh7 and Hep3B cells (Figure 4). Likewise, Isoimperatorin suppressed the expression of BCL2, survivin in survival genes, VEGF for angiogenesis and COX2 for proinflammation in Huh7 and Hep3B cells (Figure 4).

### 2.5. Isoimperatorin Inhibits c-Myc Stability in Huh7 and Hep3B Cells

**Figure 5:**
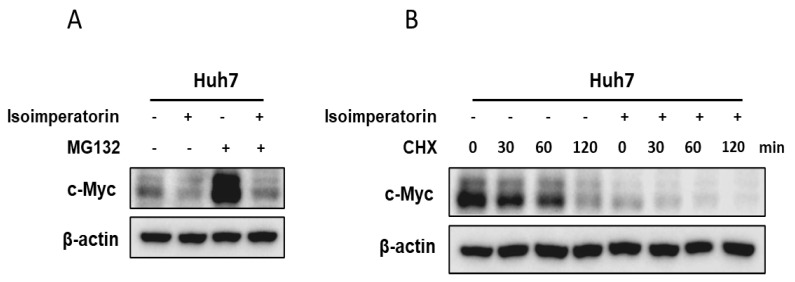
Effect of Isoimperatorin on c-Myc expression in Huh7 cells treated by MG132 or cycloheximide. (**A**) Effect of Isoimperatorin on c-Myc expression in Huh7 cells treated by MG132. Huh7 cells were treated with Isoimperatorin (60 μM) for 24 h with or without MG132 and then were subjected to Western blotting. (**B**) Effect of Isoimperatorin on c-Myc expression in Huh7 cells treated by cycloheximide. Huh7 cells were treated with Isoimperatorin (60 μM) for 24 h with or without MG132 and then were subjected to Western blotting.

The increase in c-Myc stability was induced in mitotic cells in association with inhibited c-Myc ubiquitination, since the c-Myc protein is usually degraded very rapidly within a half-life of 20 to 30 min [24]. Here, Isoimperatorin abrogated the expression of c-Myc in Huh7 cells during the exposure of the proteasome inhibitor MG132 (Figure 5A). Also, Isoimperatorin reduced the stability of c-Myc in a time-dependent manner in Huh7 cells during the exposure of the DNA synthesis inhibitor cycloheximide in Huh7 cells (Figure 5B).

### 2.6. Isoimperatorin Inhibits the Expression of c-Myc and SIRT1 via c-Myc Degradation in Huh7 Cells

**Figure 6:**
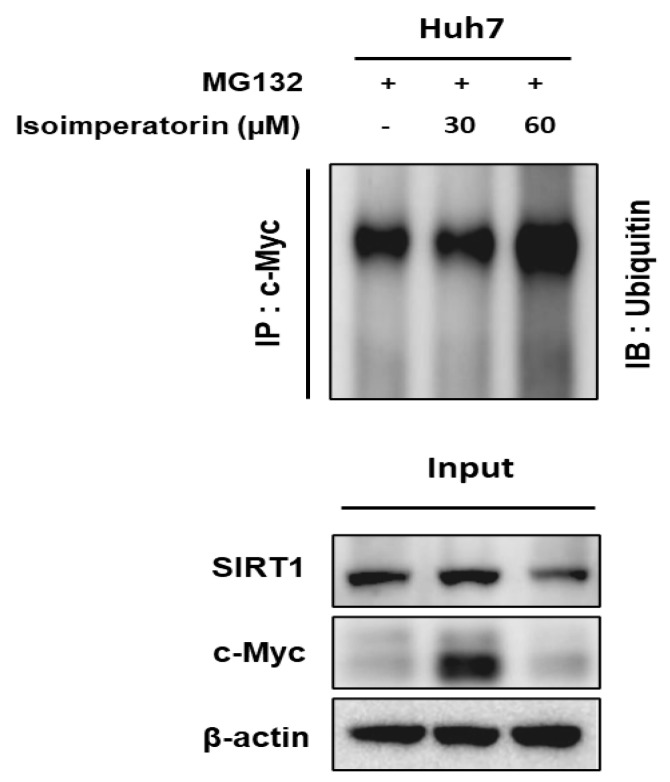
Effect of Isoimperatorin on the expression of c-Myc and SIRT1 and also c-Myc degradation in Huh cells. Huh7 cells were transfected with plasmids (V5-c-Myc and HA-Ubiquitin), followed by the addition of 20 μM of the proteasome inhibitor MG132 for 2 h, immunoprecipitated with anti-HA antibody and protein G-agarose beads, and immunoblotted with c-Myc and SIRT1.

The inhibition of proteasome activity by MG132 suppresses c-Myc degradation, since c-Myc is a substrate for ubiquitination [25]. Indeed, proteasome inhibitors including MG132 (carbobenzoxyl-leucinyl-leucinyl-leucinal-H) and bortezomib (boronic acid dipeptide derivative) have been reported to have anticancer activity [26,27]. In this study, Isoimperatorin attenuated the expression of c-Myc and SIRT1 in Huh7 cells and also enhanced the degradation of c-Myc in the presence of the proteasome inhibitor MG132 (Figure 6).

### 2.7. Isoimperatorin Disturbs the Binding between c-Myc and SIRT1 in Huh7 Cells

**Figure 7:**
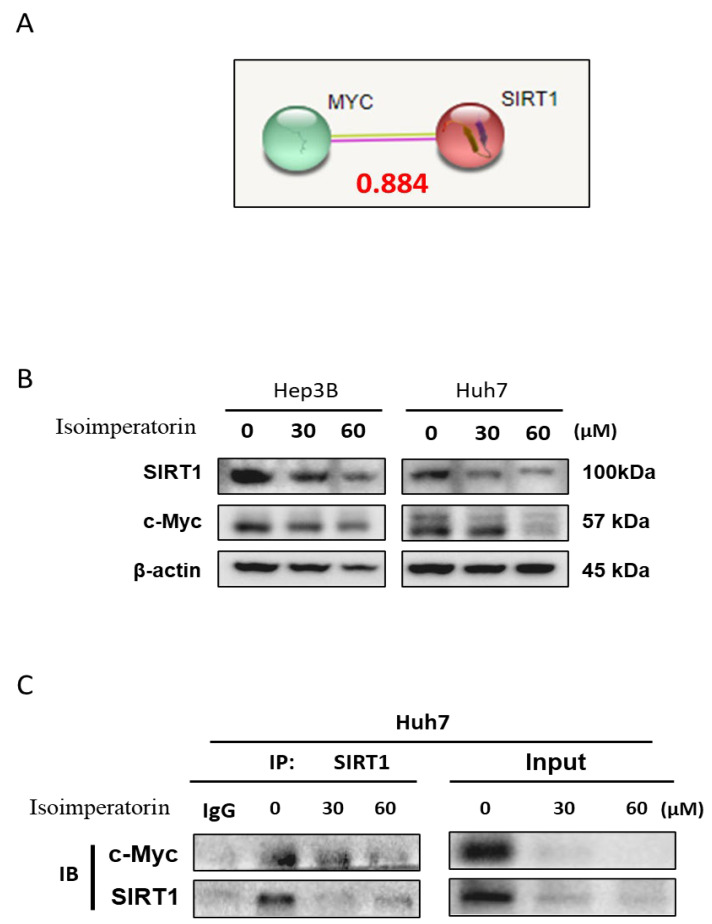
Inhibitory effect of Isoimperatorin on the binding between c-Myc and SIRT1 in Huh cells. (**A**) The binding score between c-Myc and SIRT1 using String database; (**B**) the effect of Isoimperatorin on the expression of c-Myc and SIRT1 in Huh cells; and (**C**) the effect of Isoimperatorin on the binding between c-Myc and SIRT1by Immunoprecipitation.

Isoimperatorin attenuated the expression of c-Myc and SIRT1 in Huh7 cells (Figure 7B). The binding score between c-Myc and SIRT1 was 0.884 using the String database (Figure 7A). Consistently, it was confirmed that c-Myc binds to SIRT1 in Huh7 cells by Immunoprecipitation assay (Figure 7C).

## 3. Discussion

Previous evidence revealed that Isoimperatorin was isolated and identified chemically from Angelicae dahuricae [28] and also was known to exert an antitumor effect in several cancers. Isoimperatorin induced apoptosis in SGC-7901 gastric cancer cells via the mitochondria-dependent pathway [22] and also inhibited the epithelial-to-mesenchymal transition (EMT) in colorectal and hepatocellular carcinoma cells [29]. Despite its antitumor efficacy, the underlying apoptotic mechanism remains unclear to date. Thus, the underlying apoptotic mechanism of Isoimperatorin was explored in Huh7 and Hep3B hepatocellular cancer cells.

Cell death is known to be the result of one of two distinct processes, either apoptosis (programmed cell death) or necrosis (uncontrolled cell death) [30], or autophagy-dependent cell death [31]. Apoptosis is characterized by a number of characteristic morphological changes in the structure of the cell, together with a number of enzyme-dependent biochemical processes [32]. Also, apoptotic caspases work predominantly to initiate caspases (caspase-2, -8, -9, and -10) and activate effector caspases (caspase-3, -6, and -7) [33,34]. These apoptotic caspases are further sub-grouped as initiator and effector caspases based on their order of function in the execution of apoptosis. In the current work, Isoimperatorin showed significant cytotoxicity in Huh7 and Hep3B cells. In the cell cycle analysis and Western blotting, performed to confirm that the cytotoxicity of HCCs was due to apoptosis, Isoimperatorin increased the subG1 apoptotic portion and also attenuated the expression of pro-PARP and pro-caspase 3 in Huh7 and Hep3B cells, implying the apoptotic effect of Isoimperatorin.

CDK4 and CDK6 are known to promote cell cycle progression from G0/G1 into the S phase [35,36]. Also, it is well documented that E-type cyclins (Cyclin E1 and Cyclin E2, and CCNE1 and CCNE2 genes) associated with CDK2 are known to promote G1/S transition in the cells [37]. Notably, p21 is upregulated after activation of p53 by the induction of DNA damage, leading to RB-E2F complex formation and the downregulation of cell cycle genes [38]. Here, Isoimperatorin abrogated the expression of cyclin D1, cyclin E1, CDK2, CDK4, and CDK6 and increased p21 in Huh7 and Hep3B cells, indicating that Isoimperatorin regulates G1 phase arrest-related proteins.

Recently, target therapy has become attractive in live cancer treatment with some natural compounds [39,40,41,42]. Accumulating evidence indicates that c-Myc, one of the Myc gene family, modulates cell growth, differentiation, apoptosis, angiogenesis, cell cycle, cancer progression and metabolism [7]. Also, the silent information regulator sirtuin 1 (SIRT1) protein is known as a post-translational regulator that is involved in inflammation and cancer progression [43]. Hence, targeting the epigenetic modifiers of c-Myc or SIRT1 is regarded as a good strategy for effective cancer therapy. In our work, Isoimperatorin abrogated the expression of c-Myc and SIRT1 at the protein level, and also Immunoprecipitation revealed that their binding was disturbed by Isoimperatorin in Huh7 cells, implying the inhibitory effect of Isoimperatorin on c-Myc and SIRT1. Furthermore, Isoimperatorin suppressed the overexpression of c-Myc by the proteasome inhibitor MG132 and also disturbed cycloheximide-treated c-Myc stability in Huh7 cells, indicating the critical role of c-Myc in Isoimperatorin-induced apoptosis.

Collectively, these findings highlight the novelty of the pivotal role of c-Myc and SIRT1 as molecular targets in cancer treatment [7,13], and how they are critically involved in Isoimperatorin-induced apoptosis in HCCs as potent molecular targets in liver cancer therapy. Nonetheless, further studies are still required on their bioavailability, pharmacokinetics, and potential toxicity for future clinical application.

## 4. Materials and Methods

### 4.1. Cell Culture

Huh7 and Hep3B human hepatocellular cancer cells and L-929 normal fibroblast cells were supplied by Korean Cell Line Bank (KCLB, Seoul, Republic of Korea). The cells were grown in RPMI1640 with 10% FBS and 1% antibiotic (Welgene, Gyeongsan, Republic of Korea) at 37 °C under a 5% CO_2_ incubator.

### 4.2. Cytotoxicity Assay

Based on the paper of Cho et al. [44], the cytotoxicity of Isoimperatorin (CAS 482-45-1, Merck, Darmstadt, Germany), purchased from Sigma Aldrich, was measured at the concentrations of 15, 30, 60 and 120 μM, based on 3-(4,5-dimethylthiazol-2-yl)-2,5diphenyltetrazolium bromide (MTT) assay. In brief, Huh7 and Hep3B cells (1 × 10^4^ cells/well) in a 96-well culture plate were treated with Isoimperatorin for one day and then exposed to MTT (1 mg/mL) and dimethyl sulfoxide (DMSO), followed by optical density (OD) analysis under a microplate reader at 570 nm.

### 4.3. Wound-Healing Assay

As previously described [2], Huh7 and Hep3B cells (2.5 × 10^5^ cells/well), seeded onto 12 well culture plates, were treated with Isoimperatorin for one day. Then, the scraping of the attached layers of cells was performed by a pipette tip and then the morphology was photographed by light microscope and the distance was calculated for its migratory activity.

### 4.4. Cell Cycle Analysis

Based on the paper of Jung et al. [45], Huh7 and Hep3B cells (2 × 10^5^ cells/mL) were grown with Isoimperatorin (0, 30 and 60 μM) for one day, and then were fixed in 75% ethanol. The cells were also treated by RNase A (10 mg/mL) and propidium iodide (50 μg/mL) in the dark. The DNA content of stained cells was measured by FACSCalibur with CellQuest Pro-Software 7.5.3.

### 4.5. RNA Interference

Huh7 and Hep3B cells were transfected with siRNA plasmids (control or FBW) (Bioneer, Daejeon, Republic of Korea). Then, the cells were exposed to INTERFERinTM transfection reagent for 15 min and then were maintained at 37 °C for the next molecular work.

### 4.6. Western Blotting

Based on the paper of Jung et al. [45], Huh7 and Hep3B cells exposed to Isoimperatorin (5 and 10 μM) for one day were lysed in lysis solution (1 mM Na_3_VO_4_, 1 mM NaF, 50 mM Tris–HCl, pH 7.4, 150 mM NaCl, 1% Triton X-100, 0.1% SDS, 1 mM EDTA, and 1× protease inhibitor cocktail) on ice, and centrifuged at 14,000× *g* for 20 min at 4 °C by using an RC DC protein assay kit (BIO-RAD, Hercules, CA, USA). Then, the protein samples were separated on 4–12% NuPAGE Bis–Tris gels and uploaded into a Hybond ECL transfer membrane to identify the antibodies of PARP, caspase3, BCL2, survivin, CDK2, CDK4, CDK6, p21, Cyclin D1, Cyclin E1, VEGF, COX-2, c-Myc, SIRT1 and β-actin.

### 4.7. Cycloheximide Assay

Cycloheximide assay was performed based on the paper of Aprelikova et al. [46]. Huh7 and Hep3B cells treated with Isoimperatorin (60 μM) for one day were grown with 50 μg/mL cycloheximide for different times (0, 30, 60 and 120 min) for further Western blotting.

### 4.8. Ubiquitination Assay

Huh7 cells were transfected with c-Myc and HA-Ubiquitin plasmids, followed by the addition of 20 μM proteasome inhibitor MG132 for 2 h, and then immunoprecipitated with anti-HA antibody and protein G-agarose beads. Finally, it was immunoblotted with the antibodies of c-Myc and SIRT1.

### 4.9. Co-Immunoprecipitation Assay

Based on the paper of Cho et al. [44], Huh7 and Hep3B cells treated with or without Isoimperatorin (30, 60 μM) were lysed in lysis buffer solution. The cell lysates were immunoprecipitated with the antibodies of c-Myc and SIRT1. After the cells were exposed to Protein A/G sepharose beads, the final precipitated proteins were subjected to Western blotting with the antibodies of SIRT1 and c-Myc.

### 4.10. Statistical Analysis

All data were represented by means ± standard deviation (SD). To evaluate statistical significance, the Student *t*-test was used for assessing the different significance between the control and Isoimperatorin-treated groups by using Sigmaplot version 12 software. The value of *p* < 0.05 between Isoimperatorin and the untreated control groups was determined to be statistically significant.

## 5. Conclusions

Isoimperatorin from *Angelicae dahuricae* inhibited the viability, increased the subG1 apoptotic portion and also abrogated the expression of pro-PARP and pro-caspase 3 in Huh7 and Hep3B cells. Also, Isoimperatorin abrogated the expression of cyclin D1, cyclin E1, CDK2, CDK4, and CDK6, increased p21 and reduced the expression and their binding of c-Myc and SIRT1 in Huh7 cells. Furthermore, Isoimperatorin suppressed the overexpression of c-Myc by the proteasome inhibitor MG132 and also disturbed cycloheximide-treated c-Myc stability in Huh7 cells. Taken together, it can be concluded that Isoimperatorin induced apoptosis via the pivotal role of c-Myc and SIRT1 in HCCs as a potent candidate for liver cancer therapy.

## Data Availability

All the data and materials supporting the conclusions are included in the main paper.
